# Differential Roles for EphA and EphB Signaling in Segregation and Patterning of Central Vestibulocochlear Nerve Projections

**DOI:** 10.1371/journal.pone.0078658

**Published:** 2013-10-10

**Authors:** Michelle R. Allen-Sharpley, Michelle Tjia, Karina S. Cramer

**Affiliations:** Department of Neurobiology and Behavior, University of California Irvine, Irvine, California, United States of America; University of South Florida, United States of America

## Abstract

Auditory and vestibular afferents enter the brainstem through the VIIIth cranial nerve and find targets in distinct brain regions. We previously reported that the axon guidance molecules EphA4 and EphB2 have largely complementary expression patterns in the developing avian VIIIth nerve. Here, we tested whether inhibition of Eph signaling alters central targeting of VIIIth nerve axons. We first identified the central compartments through which auditory and vestibular axons travel. We then manipulated Eph-ephrin signaling using pharmacological inhibition of Eph receptors and *in ovo* electroporation to misexpress EphA4 and EphB2. Anterograde labeling of auditory afferents showed that inhibition of Eph signaling did not misroute axons to non-auditory target regions. Similarly, we did not find vestibular axons within auditory projection regions. However, we found that pharmacologic inhibition of Eph receptors reduced the volume of the vestibular projection compartment. Inhibition of EphB signaling alone did not affect auditory or vestibular central projection volumes, but it significantly increased the area of the auditory sensory epithelium. Misexpression of EphA4 and EphB2 in VIIIth nerve axons resulted in a significant shift of dorsoventral spacing between the axon tracts, suggesting a cell-autonomous role for the partitioning of projection areas along this axis. Cochlear ganglion volumes did not differ among treatment groups, indicating the changes seen were not due to a gain or loss of cochlear ganglion cells. These results suggest that Eph-ephrin signaling does not specify auditory versus vestibular targets but rather contributes to formation of boundaries for patterning of inner ear projections in the hindbrain.

## Introduction

Inner ear neurons are grouped into multiple ganglia that transmit signals from sensory epithelia into the brainstem. In birds these epithelia include a single auditory organ and seven vestibular organs. The centrally projecting axons of these ganglion cells coalesce to form the VIIIth cranial (vestibulocochlear) nerve. Upon reaching the hindbrain, VIIIth nerve fibers diverge and project to distinct targets. In chickens, the primary contacts for auditory VIIIth nerve fibers are n. *magnocellularis* (NM) and n. *angularis* [1,2], whereas the primary vestibular fibers contact multiple hindbrain vestibular nuclei and the posterior cerebellum [3,4]. In addition, cholinergic efferent fibers travel through the VIIIth nerve to reach their inner ear targets [5-7].

As regenerative medicine advances, exciting possibilities arise to restore auditory function for deaf and hearing impaired populations [8-10], or vestibular function for those who suffer from vestibulopathies [11-13]. However, our understanding of axon guidance from the inner ear to the brain lags behind that of other sensory regions [14-16]. Studies of inner ear ganglion cell projections *in vivo* and explanted neurite projections *in vitro* have increasingly shed light on peripheral axon guidance [17,18] but the mechanisms that establish central connections have not been identified. 

In this study we address the role of Eph receptor tyrosine kinases and their membrane-associated ephrin ligands, which together comprise a large family of proteins known to regulate axon guidance. Eph-ephrin signaling can be bidirectional and mediates intercellular communication that leads to attraction, repulsion or migration [19]. Eph receptors and ephrins each contain an A and B class, with generally promiscuous binding within a class and limited cross-talk between classes [20,21]. We previously reported differential expression patterns of Eph receptors in the developing projections of VIIIth nerve fibers [22]. At embryonic stages when VIIIth nerve auditory fibers are entering the hindbrain [23], EphA4 is more highly expressed in the dorsolateral putative auditory region of the VIIIth nerve, while EphB2 is more highly expressed in the ventromedial putative vestibular region of the developing VIIIth nerve [22]. These distinct expression patterns suggested that individual Eph family proteins might differentially target growth of axons for different modalities. 

Previous studies have identified roles for Eph-ephrin signaling in the peripheral auditory and vestibular systems. Eph receptors and their ephrin ligands are expressed differentially in the inner ear of both mammals and birds during embryonic and post-natal development [24-29]. Vestibulocochlear ganglion explants can extend neurites in the presence of recombinant EphA7 and ephrin-B2, but are inhibited in the presence of EphB receptors [29,30]. In mutant mice, loss of all EphB signaling results in overshooting of peripheral spiral ganglion neuron (SGN) fibers past their hair cell targets [29], and an Eph-ephrin mediated mutual repulsion mechanism has recently been shown to differentially sort type I versus type 2 SGN afferent fibers [31]. Disruption of EphA4 causes fasciculation defects in the peripheral SGN fibers [32], and loss of ephrin-B1 signaling results in both overshooting and fasciculation defects of peripheral SGN fibers [29,32]. EphB2 mutations alter peripheral vestibular function in mice, and in addition lead to defects in midline growth of vestibular efferents [33].

In this study we tested whether the differential expression of EphA4 and EphB2 in the VIIIth nerve components acts as an instructive cue to guide axons toward auditory versus vestibular targets. Prior to entry of VIIIth nerve central projections, late migrating neural crest cells aggregate at the peripheral side of the nerve entry point [34]. Days later, VIIIth nerve axons arrive, with vestibular projections preceding auditory projections [4]. Genetic profiling has identified candidate transcription factors that specify auditory versus vestibular ganglion cell fate during development [17,35-37] but recent findings [38,39] suggest that this cell fate decision likely requires a combination of intrinsic and extrinsic signaling cues. We previously demonstrated that Eph signaling influences targeting and morphogenesis in the auditory brainstem nuclei [40-43]. Here we extend these studies to determine the role of Eph receptors and ephrins in determining the growth patterns of VIIIth nerve axons. We tested whether Eph-ephrin signaling plays a role in central pathfinding of the vestibulocochlear nerve fibers. We found that disruption of Eph-ephrin signaling did not lead to gross mistargeting into inappropriate regions. However, we found significant changes in the dimensions of hindbrain compartments through which axons travel, suggesting that Eph-ephrin signaling contributes to the establishment of compartment borders and to the dorsoventral patterning of these projection pathways.

## Methods

### Embryos

Fertilized brown Leghorn chicken eggs (*Gallus domesticus*) were purchased from AA Laboratories (Westminster, CA) and stored at room temperature prior to use, then placed in a rotating incubator at 39°C with a relative humidity above 70% to initiate development. All embryos were either electroporated at E2 and allowed to develop *in ovo*, or were transferred to a culture dish (*ex ovo*, described previously [40]) at E3 for daily recombinant protein injections from E4 – E7. Following either protocol, all embryos were maintained in a non-rotating incubator at 39°C and relative humidity above 70% and all were sacrificed at E8.

### *In ovo* electroporation

Eggs were windowed as described previously and the embryo was visualized with a small amount of 4% India ink in phosphate buffered saline (PBS; 0.4M Na_2_HPO_4_, 0.4M KH_2_PO_4_, 2.3M NaCl, pH 7.4). A fine tungsten needle was used to remove overlying membranes and sterile PBS was placed over the region of interest. Either a right-sided or bilateral transfection was performed depending on the developmental stage; only the right side was used after HH stage 14 [44] when the embryonic head was turned. Paired gold plated electrodes (Genetrodes, Harvard Apparatus) were positioned straddling the otocyst(s), approximately 3 mm apart. Plasmid DNA (1-3 µg/µL in 10mM Tris-Cl, pH 8.5) colored with a small amount of Fast Green dye, was injected into otocyst(s) using a 1.2 mm pulled glass pipette attached to a Picospritzer using a series of (typically 2-4) 20-50 ms duration injections at 20 psi. Approximately 10-20 pulse trains (12 Volts amplitude at 50-ms duration, 4-6 pulses per train with 100 ms intervals) were delivered using a BTX ECM 830 electroporator (Harvard Apparatus, Holliston, MA). Samples were included in the analysis if we could detect GFP expression in the inner ear ganglia and central VIIIth nerve projections of transfected embryos.

### Plasmids

Plasmid vectors [40,41] were introduced into otocysts using *in ovo* electroporation. Plasmids in the pMES vector contained full-length chick EphB2 (GenBank accession number NM206951.3) or EphA4 (GenBank accession number D38174), or kinase inactive forms (kiEphB2 and kiEphA4), which have point mutations in the intracellular kinase domain. The pMES construct contains a chicken β-actin promoter, a CMV-IE enhancer and also encodes a GFP reporter with an internal ribosomal entry site. As a negative control, embryos were transfected with GFP alone in a pCAX vector. Transfection was assessed by microscopic examination of GFP fluorescence or anti-GFP immunolabeling in dissected brainstems and in sectioned tissue.

### Recombinant fusion protein injections

To inhibit forward signaling, we used soluble recombinant proteins as described previously [40]. The recombinant proteins injected were EphA4-Fc, EphA3-Fc, EphB1-Fc, or IgG-Fc as a negative control (R&D Systems, catalog numbers 641-A4-200, 640-A3-200, 1596-B1-200 and 110-HG-100, respectively). Recombinant proteins were diluted to 10 µg/mL in sterile PBS and the injection solution was colorized with methylene blue powder (Sigma-Aldrich) dissolved at 37°C immediately prior to use. Small volumes of protein were pressure injected into the otocyst in whole embryo explants through a 1.2 mm pulled glass pipette attached to a Picospritzer. Injections were repeated for four consecutive days *in vitro*, corresponding with E4-E7.

### *In vitro* axon tracing

One of the goals of this study was to determine whether affecting Eph-ephrin signaling in ganglion cells would lead to mistargeting of the central auditory VIIIth nerve axons. We used a protocol we developed for injecting the basilar papilla to label the central projections of cochlear ganglion cells [45]. Small injections were used to ensure that only auditory axons were labeled and thus all experiments included only a subset of the auditory axons. Embryos in which auditory axons were cleanly traced from the cochlear ganglion into the hindbrain (n=24) were included in axon targeting analyses. Briefly, embryos were partially dissected while submerged in oxygenated artificial cerebrospinal fluid (aCSF; 130 mM NaCl, 3 mM KCl, 1.2 mM KH_2_PO_4_, 20 mM NaHCO_3_, 3 mM HEPES, 10 mM Glucose, 2 mM CaCl_2_, 1.3 mM MgSO_4_; pH 7.0) to reveal the cochlear duct of the treated side. Small injections of rhodamine dextran amine (RDA, MW 3000) were delivered with a pulled glass micropipette broken to 35µm tip and attached to a Picospritzer. Injections targeted the area directly adjacent and deep to the cochlear duct where cochlear ganglion cells and their central-projecting axons are present. Samples were incubated in aCSF for 20-30 minutes and immediately fixed in 4% paraformaldehyde (PFA) in PBS.

### Histology

Following fixation in 4% PFA for at least 2 hours, tissue was washed in PBS for 10 minutes, then cryoprotected the tissue with overnight incubation in 30% sucrose. Tissue was embedded in OCT medium and cryosectioned at 25 µm sections in the coronal plane. Alternating series of sections were collected on chrome-alum coated glass slides then dried on a 37°C slide warmer at 37°C. Some sections were counterstained with bisbenzimide (2 µg/mL in PBS for 5 min followed by 1X PBS wash for 5 min) to label cell nuclei and facilitate identification of central auditory nuclei targets. For samples that were transfected, one series was labeled with anti-GFP immunofluorescence to enhance visualization of GFP-positive axons. All samples were labeled with anti-neurofilament immunofluorescence to view axon tracts in the hindbrain. Slides were coverslipped with Glycergel mounting medium (Dako) and stored in the dark at 4°C until analyzed. 

### Immunofluorescence

Mounted sections were enclosed with a hydrophobic Pap-pen and the slides were rinsed then incubated in blocking solution (4% goat serum, 0.01% Triton in PBS) for 1 hour at room temperature in a humid chamber. The slides were quickly washed in PBS and then incubated with the primary antibody or antibodies (either anti-GFP 1:1000, anti-neurofilament heavy chain 1:500, or both) in a humid chamber at room temperature overnight. Slides were then incubated with an Alexa Fluor (Invitrogen, Carlsbad, CA) secondary antibody used at a 1:1000 dilution in blocking solution and incubated for 2 hours at room temperature in the dark.

### Image and data analysis

All samples were coded and analyzed blind to experimental conditions. The slides were viewed on a Zeiss AxioSkop-2 epifluorescence microscope and 10X objective images were taken of the hindbrain region for all sections containing the VIIIth nerve entry point. We quantified respective auditory and vestibular projection areas and dorsoventral extents of the auditory and vestibular hindbrain components as well as the right basilar papilla and cochlear ganglion areas for each image using Axiovision software to outline regions and obtain measurements based on patterns of neurofilament immunofluorescence. Volumes were subsequently calculated by summing all area measurements for each embryo and multiplying by the section thickness. Data were only collected for treated sides or control treated sides, including the right side for recombinant protein injected embryos and right, left or bilateral for transfected embryos. To estimate the surface area of the basilar papilla, we measured the segments of epithelia attached to the cochlear ganglion in each section then multiplied the sum of lengths by the section thickness. For all analyses, each embryo represented a single data point and the entire VIIIth nerve entry point region and/or the entire cochlear ganglion extent was analyzed across multiple sections.

For all analyses, samples were analyzed blind to treatment group and were decoded after data collection and grouped for comparison of means. All statistical analyses were performed with JMP software using unpaired a multivariate analysis of variance (MANOVA) followed by post-hoc testing using Dunnett’s Method with a control comparison and a significance value of p < 0.05. Representative images were taken with a Zeiss Axiocam digital camera and Axiovision software. Additional image analysis, tiling and color rendering or color merging were performed using Adobe Photoshop and figures were prepared with Adobe Illustrator.

## Results

### Auditory and vestibular hindbrain compartments characterized at the nerve entry point

**Figure 1:**
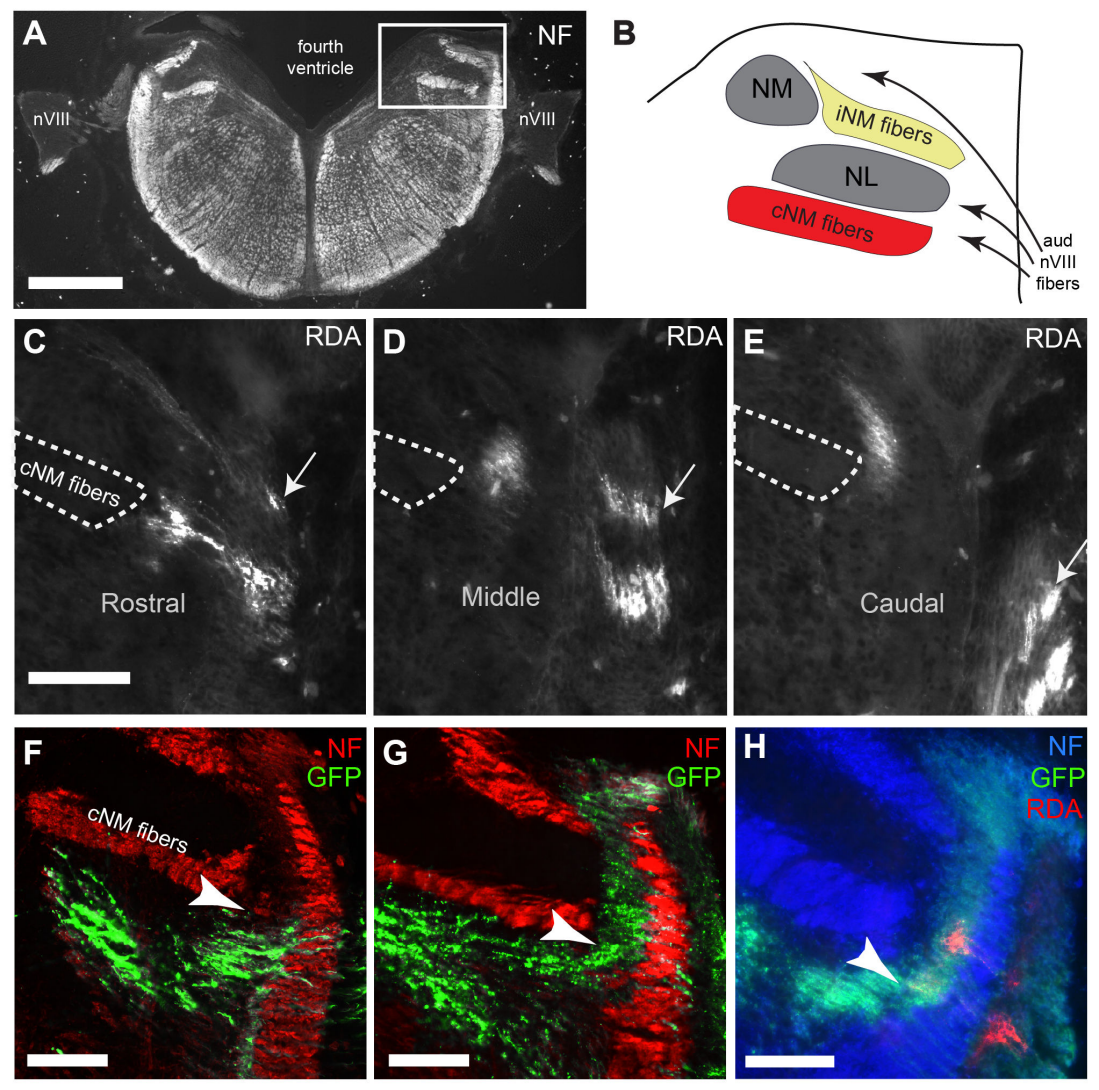
Characterization of auditory and vestibular compartments in the E8 hindbrain. (**A**) Low power image of coronal section immunolabeled for neurofilament (NF). The region containing the VIIIth nerve central projections is shown in a rectangle in upper right. Dorsal is up in all all figures. (**B**) Schematic diagram showing the key features in the boxed region in A. Neurofilament reveals the ipsilateral n. magnocellularis projection (iNM) and the contralateral projection (cNM), seen ventral to n. laminaris (NL). (**C**-**E**) Selective labeling of high and low frequency regions of cochlear ganglion cell fibers with RDA reveals the auditory extent of the hindbrain at the level of VIIIth nerve entry. The traced high frequency axons (arrow) are dorsal to the traced lower frequency axons and can be visualized in the hindbrain across several coronal sections (labeled rostral, middle, caudal). (**F**) Co-staining with neurofilament antibody demonstrates an example in which GFP transfected axons are found only in vestibular ganglion cells. NM-NL fiber tract (cNM fibers, outlined in A-C) acts as a landmark, and is used to demarcate between auditory and vestibular compartments (arrowheads). (**G**) Example in which both auditory and vestibular ganglion cells are transfected. (**H**) Embryo with both auditory and vestibular ganglion axons transfected with GFP and subjected to RDA tracing. Central RDA label shows the location of a subset of auditory fibers, which falls within characterized auditory compartment. Scale bars in A, 500 µm; in C (applies to D,E), F, G, and H, 100 µm.

We first determined the normal central trajectories of auditory and vestibular ganglion cell axons. Several complementary approaches were used, and together they revealed distinct hindbrain compartments through which auditory or vestibular axons approach their initial targets from the VIIIth nerve entry point (Figure 1). An overview of the anatomical regions are shown in Figure 1A,B. Central projections of auditory VIIIth nerve fibers could be followed into the hindbrain after injection of RDA into limited regions of the cochlear ganglion in control embryos (Figure 1C-E). Using an independent approach, otocyst transfection with GFP led to fluorescence visible in ganglion cell bodies and their centrally projecting axons. This labeling allowed us to determine which ganglion cells were transfected and which region(s) in the hindbrain contained GFP expressing axons (Figure 1F-H) as well as where RDA labeled auditory axons fell within those VIIIth nerve axons (Figure 1H). 

Together with results from previous dye-labeling studies [23], our RDA tracings of untreated or control embryos and GFP visualization in transfected ganglion cells of control embryos correlated with stereotyped axon tracts discernible with neurofilament immunofluorescence. We thus used these characteristic patterns seen with neurofilament immunofluorescence to ascertain the locations of auditory and vestibular axon tracts in all subsequent measurements. At the level of VIIIth nerve entry, the dorsolateral-most region of the hindbrain contains auditory fibers. Vestibular fibers are present more ventromedially and project in a wedge-shaped pattern with the dorsal fibers extending most medially. Central auditory projections traveled into the hindbrain with robust borders that we never observed within the ventral region containing the majority of vestibular projections. Thus, we used the ventrolateral corner of a thick central fiber tract arising from NM to n. *laminaris* (NL) second and third order auditory neurons, as a landmark to provide a linear demarcation between the two compartments since it is reliably situated at this transitional area (arrowheads in Figure 1F-H).

### Auditory axons remain in auditory central regions when Eph signaling is blocked

**Figure 2:**
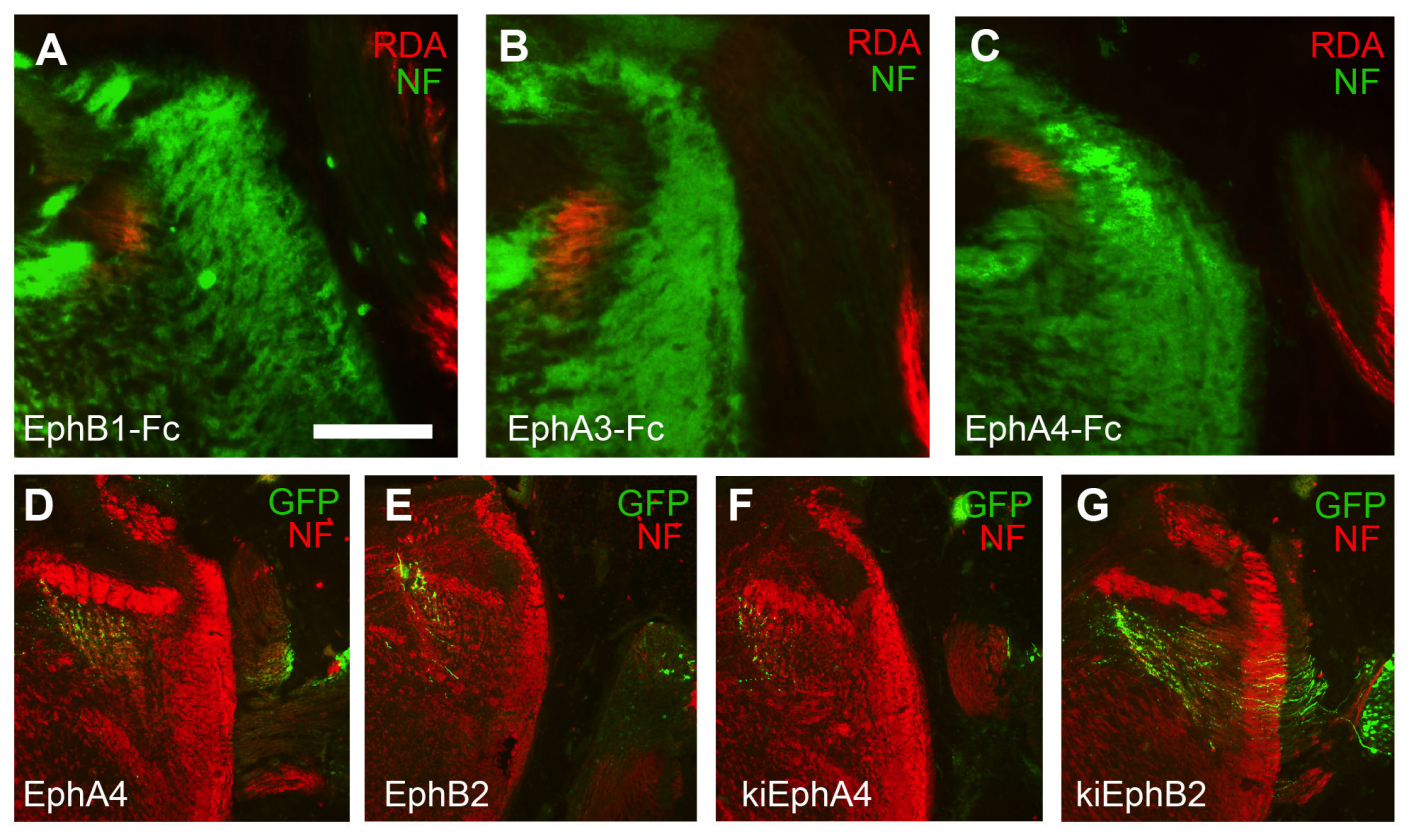
Eph receptor inhibition does not alter auditory vs. vestibular targeting. (**A**) RDA tracing combined with neurofilament immunofluorescence was used to determine whether auditory axons were correctly targeted. An example of EphB1-Fc treated embryo with normal targeting is shown. (**B**) Example of EphA3-Fc treated embryo with normal auditory targeting. (**C**) Similarly, treatment with EphA4-Fc did not alter central targeting of auditory VIIIth nerve axons. (**D**) Targeting of vestibular axons was determined in embryos that showed GFP transfection in vestibular, but not auditory, ganglion cells. Axons were followed through brainstem sections. Transfection in this example was with EphA4; all GFP labeled axons were found in vestibular projection regions. (**E**) Transfection with EphB2 plasmid did not alter central vestibular VIIIth nerve projections. (**F**) Misexpression of kiEphA4 did not alter central vestibular projections. (**G**) Similarly, kiEphB2-transfected embryos showed no mistargeting of vestibular axons into auditory projection regions. Scale bar, 100 µm in A-C and 300 µm in D-G.

We used two complementary approaches to test whether Eph-ephrin signaling is necessary for differential targeting of the VIIIth nerve auditory and vestibular components. We broadly inhibited the Eph receptor subclasses in developing *ex ovo* preparations using Fc-fusion proteins, and we selectively misexpressed EphA4 and EphB2 receptors in inner ear ganglion cells and their projections using *in ovo* plasmid electroporation. To assess targeting of auditory axons in treated embryos, we anterogradely labeled auditory fibers by injecting the basilar papilla with RDA. Centrally projecting auditory VIIIth nerve axons were selectively traced in a total of 14 embryos (2-6 embryos per group, average of 22 [range 7 – 35] axons per embryo). Misrouting of auditory axons into the vestibular compartment (Figure 2A-C) was not observed in any of the treatment conditions. 

### Vestibular axons remain in vestibular central regions when Eph signaling is blocked

We were able to assess the targeting of vestibular axons in GFP transfected embryos in which vestibular, but not cochlear, ganglion cells were transfected (n = 16). In all of these cases, GFP labeled axons were seen only in the central vestibular projection compartments described above and not in the central auditory projections compartments (Figure 2D-G). No embryos were transfected only in the cochlear ganglion and not in vestibular ganglia.

### Vestibular hindbrain compartment is reduced following inhibition of Eph-ephrin signaling with EphA4-Fc

**Figure 3:**
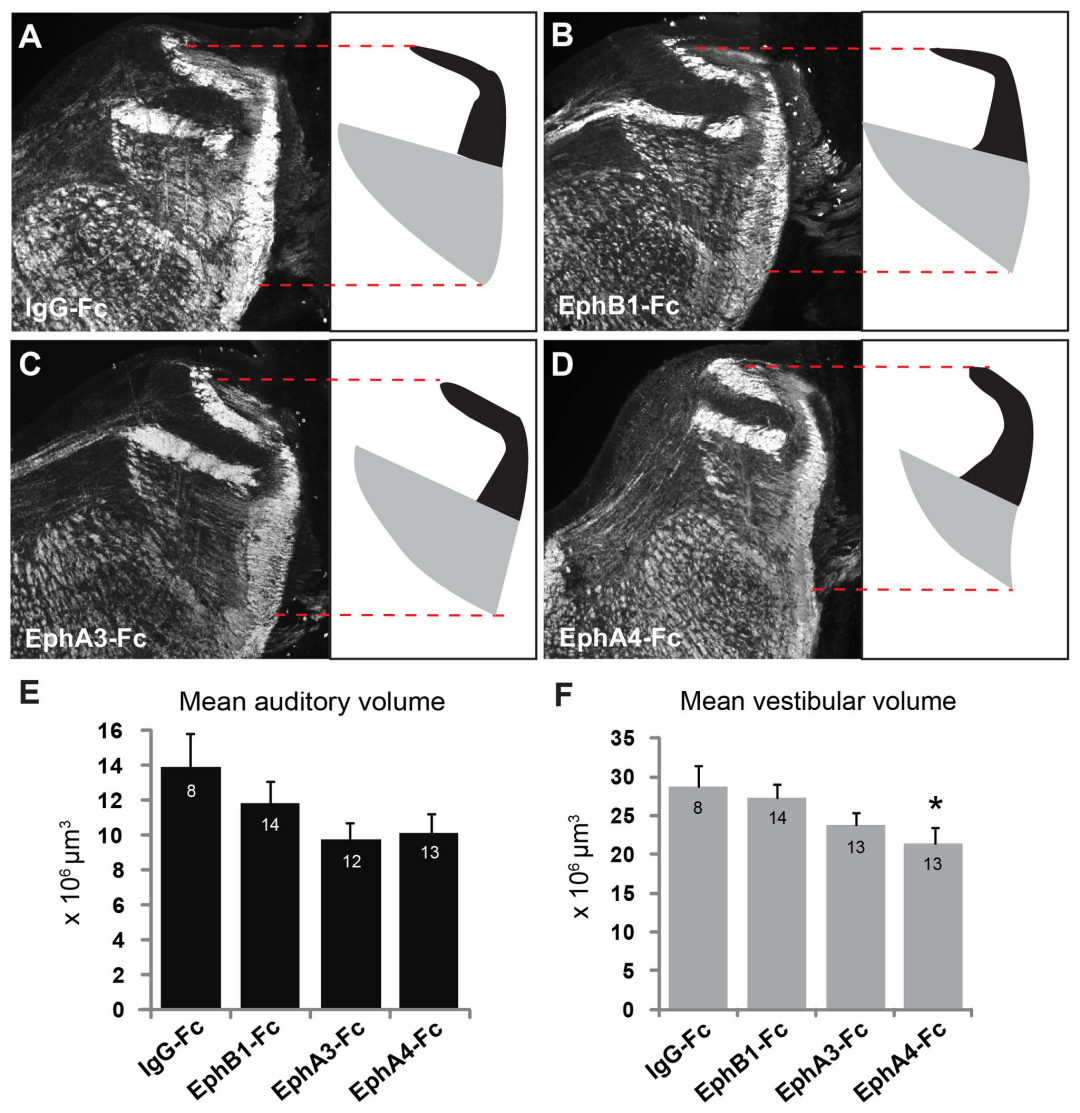
Eph receptor inhibition causes reduction in hindbrain compartment. (**A**-**D**) Neurofilament immunofluorescence (left) of hindbrain sections at level of VIIIth nerve entry with auditory (black) and vestibular (gray) components schematized (right). Red dashed line indicates entire dorsoventral extent of the VIIIth nerve projections in hindbrain. Volumes found in IgG-Fc (**A**) EphB1-Fc (**B**), and EphA3-Fc (**C**) did not significantly differ. (**D**) Vestibular hindbrain volume is reduced following EphA4-Fc treatment. (**E**,**F**) Graphs of mean auditory (**E**) and vestibular (**F**) volumes for all treatment groups. Numbers of samples are indicated in each bar; asterisk indicates p <0.05.

Axon tracing and GFP transfection studies revealed no gross mistargeting of VIIIth nerve axons into regions of inappropriate modality. We next determined whether inhibition of Eph-ephrin signaling led to changes in the formation of these central auditory and vestibular projection compartments. In the IgG-Fc control group, the mean volume of auditory projections into the hindbrain was 13.89 x 10^6^ μm^3^ ± 1.96 x 10^6^ μm^3^ (n = 8, Figure 3A). This volume did not differ significantly from those obtained after treatment with EphB1-Fc (11.81 x 10^6^ μm^3^ ± 1.27 x 10^6^ μm^3^; n = 14, p = 0.55, Figure 3B), EphA3-Fc (9.74 x 10^6^ μm^3^ ± 0.98 x 10^6^ μm^3^; n = 12, p = 0.11, Figure 3C), or EphA4-Fc (10.09 x 10^6^ μm^3^ ± 1.13 x 10^6^ μm^3^; n = 13, p = 0.14, Figure 3D) using MANOVA and post-hoc Dunnett’s method with control. In contrast, blocking Eph signaling with EphA4-Fc resulted in a significant 25% reduction of the vestibular VIIIth nerve projection volume in the hindbrain compared to IgG-Fc controls. Mean volume was 28.74 x 10^6^ μm^3^ ± 2.71 x 10^6^ μm^3^ in IgG-Fc controls (n = 8, Figure 3A) and 21.42 x 10^6^ μm^3^ ± 2.06 x 10^6^ μm^3^ following treatment with EphA4-Fc (n = 13; F = 5.0, p = 0.04, Figure 3D). Group data for auditory and vestibular compartment volumes are shown in Figure 3E and 3F, respectively.

In addition to broad treatment with fusion proteins, we performed focal transfection in E2 embryos to determine the effects of individual Eph proteins. Transfection of inner ear ganglion cells and their axons with either EphA4, EphB2, kiEphA4, or kiEphB2 did not have any significant effect on VIIIth nerve projection volumes (data not shown). 

### Peripheral kiEphA4 and kiEphB2 transfection affects dorsoventral patterning of auditory and vestibular axon tracts in the hindbrain

**Figure 4:**
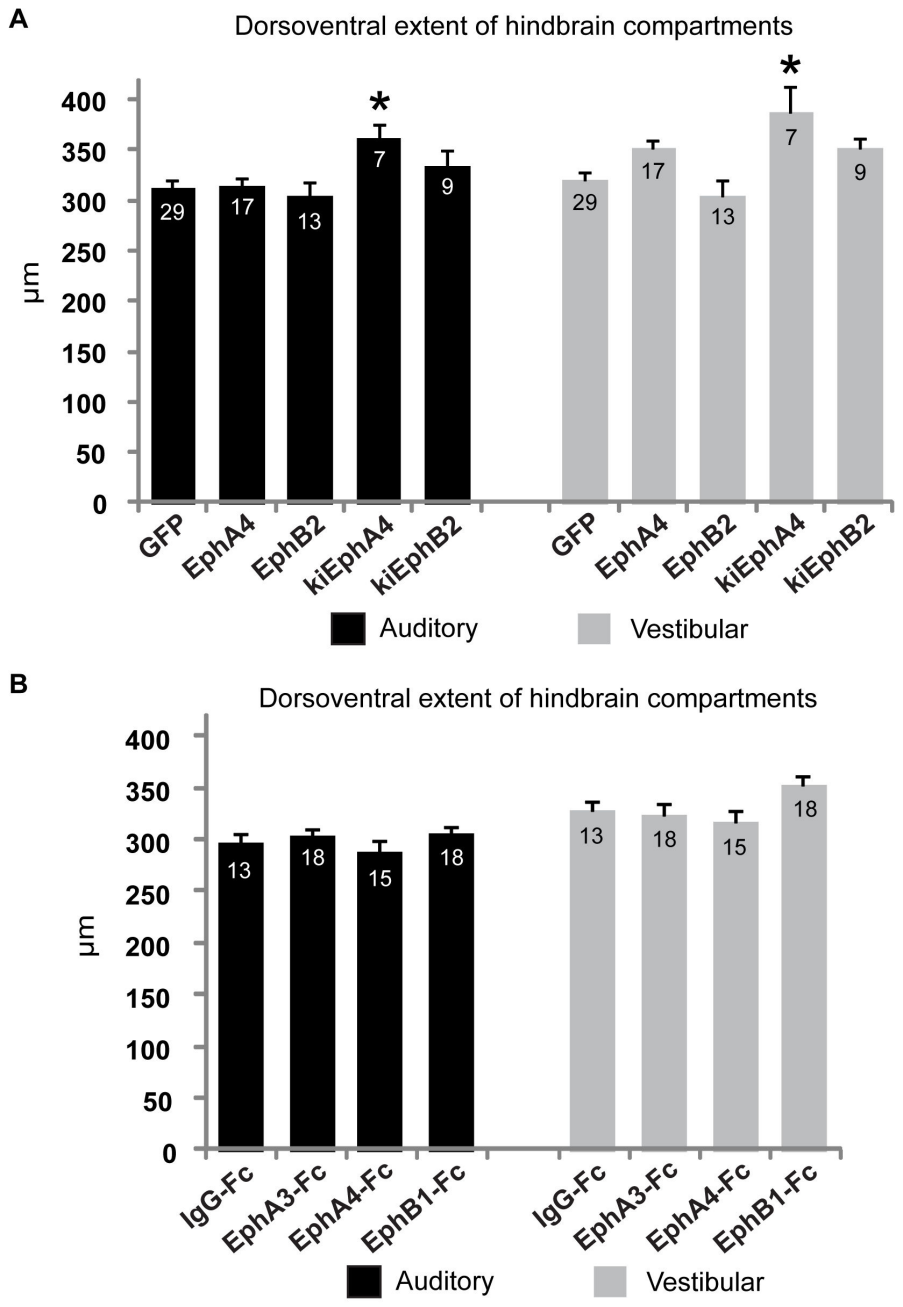
Misexpressing Eph receptors results in dorsoventral pattern changes of the central VIIIth nerve projections. Mean dorsoventral extent of auditory (black) and vestibular (gray) hindbrain compartments are shown for (**A**) transfected embryos and (**B**) Fc-fusion protein injected embryos. There was a significant increase in both compartments observed in kiEphA4 expressing embryos.

Several factors contribute to the establishment of auditory and vestibular projection volumes. As these regions are segregated along the dorsoventral axis, we analyzed the dimensions along this axis in transfected animals. The auditory and vestibular components are segregated upon entering the hindbrain, with the vestibular VIIIth nerve projections found ventral and adjacent to a large auditory fiber tract containing projections NM to contralateral NL. We found that transfection with kiEphA4 resulted in a 15% increase in the dorsoventral extent of the auditory VIIIth nerve projection, and a 21% increase in the vestibular projection. GFP controls had a mean dorsoventral extent of 312.59 μm ± 7.40 μm (n = 23) for auditory VIIIth nerve projections and 320.04 μm ± 8.92 μm (n = 23) for vestibular VIIIth nerve projections; kiEphA4 expressing embryos had a mean dorsoventral extent of 360.90 ± 15.31 μm (n = 7; p = .02) and 387.06 μm ± 25.97 μm (n = 7; p = 0.004) for auditory and vestibular projections, respectively, as shown in Figure 4A. These changes were not seen in embryos with VIIIth nerve axons overexpressing full-length wild type EphA4, suggesting that the loss of forward EphA4 signaling in the kiEphA4 transfected embryos is responsible for the effect,. A similar analysis performed in embryos with recombinant protein injections did not reveal changes in dorsoventral extent (Figure 4B), suggesting that this effect may be driven cell-autonomously by the VIIIth nerve axons. 

**Figure 5:**
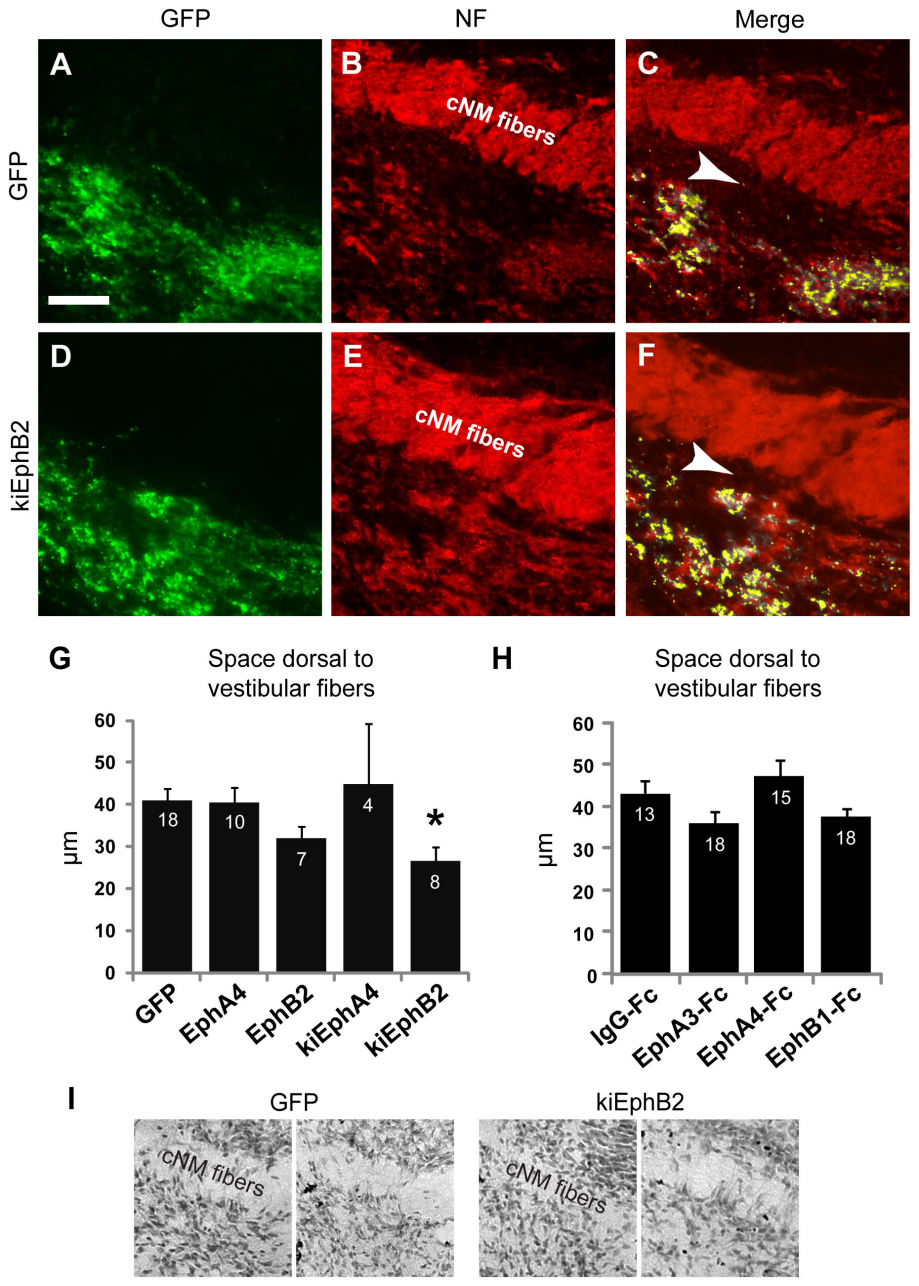
Misexpression of Eph receptors caused a change in axon tract spacing. (**A**-**F**) Vestibular VIIIth nerve axons, seen best with GFP signal (**A**, **D**) are ventral to a thick central auditory fiber tract (cNM fibers) seen best with neurofilament immunostaining (**B**, **E**). The space between these two fiber tracts (arrowhead) that is visible in GFP control embryos (**A**-**C**) is significantly reduced in kiEphB2 expressing embryos (**D**-**F**). Group means shown for electroporated embryos (**G**) and Fc-fusion protein injected embryos (**H**). (I) Nissl stain of two different GFP expressing and two different kiEphB2 expressing embryos. There is no change in cellular density ventral to the axon tract (cNM fibers). Samples shown demonstrate fiber tract separation larger than (for GFP) and smaller than (for kiEphB2) group averages to demonstrate that the axon spacing is unrelated to cellular density. Dorsal is up.

As auditory and vestibular projection regions reside in distinct dorsal and ventral locations, respectively, we tested whether Eph-ephrin signaling has a role in establishing the spacing between auditory and vestibular projection areas. In kiEphB2-transfected embryos, the axon-free space separating the contralateral NM fiber tracts and the vestibular projection was reduced by 35% compared to GFP controls. Controls had a mean distance of 41.01μm ± 2.93 μm (n = 18; Figure 5A-C); kiEphB2 transfected embryos had a mean of 26.56 μm ± 3.40 μm (n = 8, p = .047, Figure 5D-F). The same effect was not seen with full-length EphB2 transfection, suggesting that loss of forward signaling underlies this effect (Figure 5G). Again, the same effect was not found in any of the *ex ovo* fusion protein injected embryos (Figure 5H) and there were no changes to cellular density in this region (Figure 5I) to account for the reduced distance. 

### Selective inhibition of EphB signaling altered morphology of the sensory epithelium with no change in cochlear ganglion volume or hindbrain patterning

**Figure 6:**
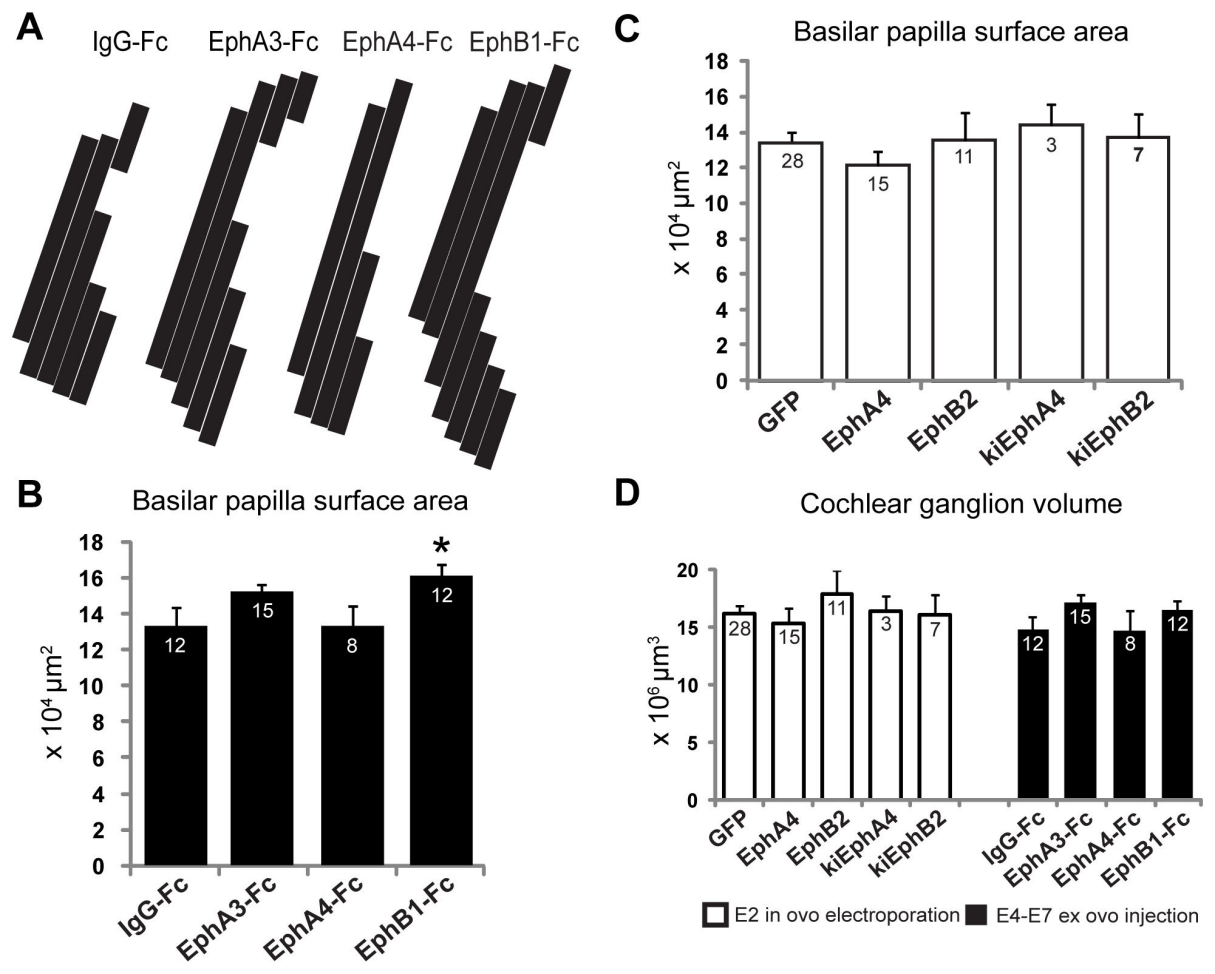
Inhibition of EphB receptors affects sensory epithelium development. (**A**) 2D reconstruction of basilar papilla surface area from measurements taken in coronal hindbrain sections. Each longitudinal stripe represents the length of basilar papilla measured in a single section, and stripes are scaled to be 50 μm in width (the thickness between sections) in order to provide an accurately scaled reconstruction. (**B**,**C**) Quantification of mean surface area for embryos that were (**B**) injected with Fc-fusion proteins from E4-E7 or (**C**) transfected at E2. (**D**) Cochlear ganglion volumes were not significantly different between any treatment groups.

Because embryos were treated at the auditory periphery, we sought to determine whether the central effects we observed could potentially have occured secondarily to changes in the periphery. We measured the surface area of the basilar papillae and the volume of the cochlear ganglia to determine whether any central effects were correlated with peripheral changes. We found that selective inhibition of EphB signaling appeared to alter basilar papilla development (Figure 6A,B). After injection of EphB1-Fc into the otic duct, the mean surface area of auditory sensory epithelium with EphB1-Fc treatment was 16.10 x 10^4^ μm^2^ ± 0.68 x 10^4^ μm^2^ (n = 12), which was significantly increased by 17% compared to IgG-Fc controls with a mean surface area of 13.34 x 10^4^ μm^2^ ± 1.02 x 10^4^ μm^2^ (n = 12; p < 0.05). These effects were not seen following treatment with EphA3-Fc (mean surface area 1.53 X 10^4^ μm^2^; n = 15, p = 0.19) or EphA4-Fc (mean surface area 13.29 x 10^4^ μm^2^ ± 0.92 x 10^4^ μm^2;^ n = 8, p = 1.0). We found no effect on the basilar papilla when VIIIth nerve axons misexpressed Eph receptors (Figure 6C) and the cochlear ganglia under all conditions were of equivalent size and volume (Figure 6D). During our dye injection procedure for axon tracing, we frequently observed that embryos in the EphB1-Fc treated group had morphologically abnormal basilar papillae that were difficult to identify for injection. Although the basilar papilla appears developmentally abnormal in the EphB1-Fc treated group compared to all other treatment conditions, there was no detectable change to the ganglion cell volume or central projection patterning, suggesting a unique requirement for EphB signaling at the periphery that may be independent of central patterning. These observations, together with the lack of effect of other fusion proteins, also suggest the central effects we observed arise directly from the interactions of VIIIth nerve axons within the hindbrain. 

## Discussion

The goal of this study was to determine the role of Eph-ephrin signaling in the guidance of VIIIth nerve axons to their central targets. Our results suggest that these signaling molecules do not by themselves regulate auditory vs. vestibular axon targeting. Instead, Eph family proteins appear to have more subtle roles in the designation of central projection boundaries and their extents. These hindbrain compartments for auditory and vestibular central VIIIth nerve projections may in turn contribute to axon navigation during circuit formation.

### Patterning of VIIIth nerve projections in the hindbrain is controlled by multiple distinct Eph-ephrin signals

Only with pharmacologic inhibition of Eph receptor signaling did we see a shift in relative amount of space within the brainstem dedicated to vestibular axons. Following treatment with EphA4-Fc, which binds strongly to ephrin-A’s and ephrin-B2 [20] the vestibular compartment was significantly reduced in size. Consistent with expression patterns found at the nerve entry point, these findings support that EphA4 signaling is required to appropriately segregate the different components of VIIIth nerve fibers. The change seen in projection volume could reflect a change in absolute number of VIIIth nerve fiber components, changes in fasciculation or distribution of fibers within the respective compartment, or a combination of these effects. The observation that ganglion volumes were similar in all treatment groups suggests that this effect results from alterations in fiber distribution within compartments. 

In contrast to results obtained using broad inhibition of Eph proteins, misexpression of wild type and kinase inactive forms of EphA4 and EphB2 receptors in the VIIIth nerve projections did not affect hindbrain compartment volumes. This discrepancy might reflect experimental differences in the effectiveness of treatment, as the pharmacological treatment acts on multiple targets and may reach a greater spatial extent than focal electroporation. Moreover, pharmacological treatment could result in cell-autonomous as well as non-cell-autonomous functions in central VIIIth nerve projections. The combined action of several Eph family proteins may thus be needed to determine projection volumes. KiEphA4-transfected embryos showed an expansion of the normal dorsoventral borders without a change in overall volume, suggesting that forward signaling through EphA4 influences the dimensions of the central pathway taken by the nerve projections. In embryos overexpressing the dominant negative kiEphB2, a reduction of normal spacing between the two fiber tracts was observed, suggesting that EphB2 maintains this dorsoventral spacing during hindbrain development. The separation between tracts may arise from chemorepulsive cues mediated through EphB2, which repels NM axons at the midline in chicks [42] and influences midline crossing of vestibular efferents in mice [33]. Separation of these two fiber tracts during early development may be an important variable in axon navigation. These misexpression studies demonstrate the contributions of individual Eph family members to the formation of auditory and vestibular projection regions.

### Potential mechanisms by which Eph-ephrin contributes to central projection regions

Although we did not observe mistargeting of auditory or vestibular axons to inappropriate target regions, we did observe significant changes to the central hindbrain compartments devoted to central VIIIth nerve fibers. The differential expression of EphA4 and EphB2 at the nerve entry point may thus reflect a mechanism that helps determine boundaries of fiber tracts; these tracts may facilitate axon navigation using additional guidance cues to direct axons to their hindbrain targets. Contrary to what is seen up to the nerve entry point, central vestibular projections strongly express EphA4 [46,47]. The reduction of vestibular hindbrain compartments following EphA4-Fc treatment is consistent with a role for EphA4 in designating a border for this pathway. An interesting possibility is that Eph expressing peripheral glia outside the nerve entry point provide guidance cues for growing VIIIth nerve axons [48]. Co-culture studies showed cochlear ganglion neurons could extend neurites along either Schwann cells or oligodendrocytes, and demonstrate a preference correlating with whichever glial cell type was in contact with the ganglion cell body [49], raising the possibility that axon navigation cues are designated locally. 

### Development of sensory epithelium is only affected by loss of EphB signaling

The extent of the auditory sensory epithelium was increased after EphB inhibition with EphB1-Fc. Previous expression studies have shown that ephrin-B1 is highly expressed in the peripheral projections of SGN cells, whereas EphB receptors are differentially expressed in subpopulations of SGN cells and hair cells [29,30]. EphB signaling may be important for regulating normal development of the basilar papilla, perhaps by limiting the extent of epithelial growth of the sensory epithelium. While other Eph family proteins are also expressed in the inner ear [25,28,32], we observed significant changes in basilar papilla length only when signaling through the EphB class was broadly inhibited, suggesting that several EphB proteins may have cooperative function in the ear. Alteration of the basilar papilla was not associated with changes in ganglion cell number or central projections. Our analysis of peripheral structures was limited to the basilar papilla, but changes in vestibular organs might be consistent with the role of EphB2 in mammalian vestibular function [33,50], where it regulates endolymphatic ion balance. 

Previous studies of central auditory axon guidance have demonstrated that Eph-ephrin signaling is needed to direct axons to appropriate regions with a target [40,41,43,51]. Additionally, EphB2 appears to inhibit midline axon growth in both auditory and vestibular axons [33,42]. Taken together, Eph-ephrin signaling instructs a wide array of processes for both auditory and vestibular development. While some functions are similar, our data suggest that these molecules are not sufficient to define modality-specific trajectories. 

## Conclusions

Auditory and vestibular afferents enter the brainstem through the VIIIth cranial nerve and reach their appropriate central targets. We tested the role of Eph family proteins in establishing these pathways. We inhibited Eph signaling using several approaches and examined auditory and vestibular ganglion cell projections in the brainstem. We found no evidence of mistargeting to inappropriate modality regions. We found instead that inhibition of EphA and EphB receptors reduced the volume of the putative vestibular projection compartment. Misexpression of kinase inactive forms of EphA4 and EphB2 in ganglion cells suggest that these central patterning effects are not cell-autonomous. They further show that EphA4 and EphB2 contribute to the dimensions and separation of the auditory and vestibular central projections. Central changes were not due to a gain or loss of cochlear ganglion cells. These results suggest that Eph-ephrin signaling does not specify auditory versus vestibular targets *per se*, but rather contributes to formation of boundaries for patterning of inner ear projections in the developing hindbrain. 
